# Quantifying ventilation by X-ray velocimetry in healthy adults

**DOI:** 10.1186/s12931-023-02517-z

**Published:** 2023-08-30

**Authors:** Trishul Siddharthan, Kyle Grealis, Jason P. Kirkness, Tamás Ötvös, Darko Stefanovski, Alex Tombleson, Molly Dalzell, Ernesto Gonzalez, Kinjal Bhatt Nakrani, David Wenger, Michael G. Lester, Bradley W. Richmond, Andreas Fouras, Naresh M. Punjabi

**Affiliations:** 1 Division of Pulmonary, Critical Care, and Sleep Medicine, University of Miami, Miami, FL USA; 2 4DMedical, Melbourne, Australia; 3 University of Pennsylvania, Philadelphia, Pennsylvania USA; 4 Division of Allergy, Pulmonary, and Critical Care Medicine, Vanderbilt University School of Medicine, Nashville, TN USA; 5 Department of Veterans Affairs Medical Center, Nashville, TN USA; 6 Department of Cell and Developmental Biology, Vanderbilt University, Nashville, TN USA

**Keywords:** X-ray velocimetry, Functional lung imaging, Ventilation heterogeneity

## Abstract

**Rationale:**

X-ray velocimetry (XV) has been utilized in preclinical models to assess lung motion and regional ventilation, though no studies have compared XV-derived physiologic parameters to measures derived through conventional means.

**Objectives:**

To assess agreement between XV-analysis of fluoroscopic lung images and pitot tube flowmeter measures of ventilation.

**Methods:**

XV- and pitot tube-derived ventilatory parameters were compared during tidal breathing and with bilevel-assisted breathing. Levels of agreement were assessed using the Bland-Altman analysis. Mixed models were used to characterize the association between XV- and pitot tube-derived values and optimize XV-derived values for higher ventilatory volumes.

**Measurements and main results:**

Twenty-four healthy volunteers were assessed during tidal breathing and 11 were reassessed with increased minute ventilation with bilevel-assisted breathing. No clinically significant differences were observed between the two methods for respiratory rate (average Δ: 0.58; 95% limits of agreement: -1.55, 2.71) or duty cycle (average Δ: 0.02; 95% limits of agreement: 0.01, 0.03). Tidal volumes and flow rates measured using XV were lower than those measured using the pitot tube flowmeter, particularly at the higher volume ranges with bilevel-assisted breathing. Under these conditions, a mixed-model based adjustment was applied to the XV-derived values of tidal volume and flow rate to obtain closer agreement with the pitot tube-derived values.

**Conclusion:**

Radiographically obtained measures of ventilation with XV demonstrate a high degree of correlation with parameters of ventilation. If the accuracy of XV were also confirmed for assessing the regional distribution of ventilation, it would provide information that goes beyond the scope of conventional pulmonary function tests or static radiographic assessments.

**Supplementary Information:**

The online version contains supplementary material available at 10.1186/s12931-023-02517-z.

## Introduction

Over the last decade, substantial progress has been made in the development of new approaches to pulmonary imaging. X-ray velocimetry (XV) is a novel quantitative method which utilizes fluoroscopic lung images to provide measurements of lung motion and regional ventilation [1–4]. XV uses cross-correlation functions calculated from fluoroscopic lung images acquired at multiple viewing angles at sequential time points to calculate the displacement, and therefore the velocity, of a particular region of the lung [5]. It was first utilized to characterize regional ventilation in an animal model of bleomycin toxicity to demonstrate the correlation between lung structure and function [6]. Histological changes observed in different portions of the bleomycin-treated lungs were correlated to regional impairments of lung expansion. Moreover, physiologically relevant abnormalities in distribution of ventilation were evident with XV well before abnormalities in plethysmography-derived lung compliance and tidal volume measurements. XV has also been used in assessing regional filling defects of the lung in an animal of cystic fibrosis, as well as models of explanted lungs with emphysema [7, 8]. Using the βENaC transgenic mice model, XV was used to demonstrate areas of reduced expansion and alterations in the time constant corresponding to histologically abnormal sections of the lung resulting from mucus obstructing the bronchial airways [9]. Thus, XV provides an anatomical assessment of regional ventilation abnormalities and yields unique insights not available with conventional pulmonary function or radiographic testing.

Given that XV-derived measures of regional ventilation correlate well with histopathological changes in preclinical models, XV holds immense potential for characterizing the physiological impairments in disorders that affect the lung. The pathological heterogeneity that is common to disorders of the lung results in regional differences in its static and dynamic properties. Such functional heterogeneity is not captured with conventional pulmonary function testing. To assess the value of XV in pulmonary medicine, this study was designed to assess whether XV analysis of fluoroscopic lung images can be used to quantify parameters of ventilation including tidal volume, and airflow in healthy adults. Secondary outcomes included respiratory rate and duty cycle. Assessment of the agreement of global parameters is a starting point that encourages further studies assessing the validity of the technique for assessing the regional distribution of ventilation. Such assessments would help advance the understanding of the pathophysiology of obstructive and restrictive lung disease and associated effects of various treatment strategies.

## Methods

Twenty-four healthy adults were recruited from the general community. Written informed consent was obtained for all participants and the study was approved by the University of Miami IRB. To exclude prevalent comorbidity, each participant completed a demographics questionnaire and pulmonary function testing including spirometry, body plethysmography, and measurement of diffusing capacity of the lung. The protocol included determination of respiratory rate, tidal volume, and airflow using a pitot tube flowmeter under conditions of quiet tidal breathing [10]. A subset of the volunteers (N = 11) were also assessed with assisted ventilation using a non-invasive bilevel positive airway pressure device (Resmed S8 Autoset, San Diego). The decision to assess ventilatory parameters under the two conditions was made to provide a wide range of ventilatory volumes and airflows.

**Fig. 1:**
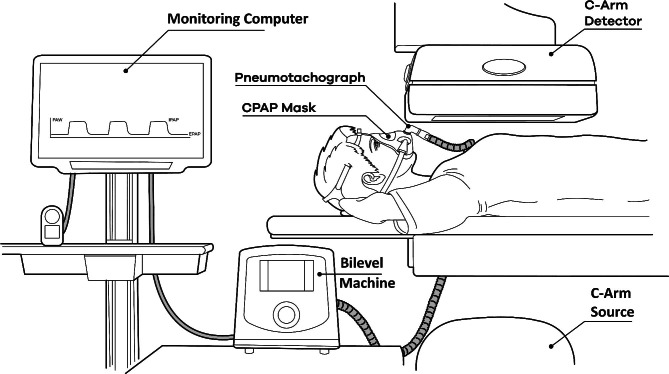
Schematic of the experimental setup illustrating the breathing circuit used for monitoring spontaneous tidal breathing and bilevel assisted breathing along with position of the fluoroscopy C-arm source and detector

Each participant was fitted with a leak-free ventilation full-face mask (ResMed AirTouch, San Diego) attached to a pitot tube airflow meter and an inline exhalation port as previously described [10]. For the bilevel component of the study protocol, a two-meter air tube was connected to S8 autoset (ResMed, Australia) in spontaneous-time mode with an expiratory positive airway pressure of 5 cm H_2_O, an inspiratory positive airway pressure of 15 cm H_2_O and a backup rate of eight breaths per minute. Under tidal and bilevel-assisted breathing in the supine position, fluoroscopic lung images were obtained sequentially using C-arm fluoroscopy at five distinct angles across participant’s chest with the same center of rotation as follows: 0° PA (Posterior-Anterior axis), ± 36° from PA, and ± 72° from PA. Imaging sequences were acquired at 15 fps capturing at least one complete, continuous tidal breath. The subject remained in the same position for all imaging sequences (Fig. 1). Velocimetry Lung Ventilation Analysis Software (4DMedical Limited, Australia) was utilized to derive respiratory rate, duty cycle, tidal volume, and airflow by analysis of the fluoroscopic images. Concurrent measurements were also obtained from the pitot tube flow meter (See Online Supplement).

### Statistical analysis

The following parameters were derived from the XV and pitot tube analysis of the fluoroscopic images: respiratory rate, inspiratory time (T_insp_), total breath time (T_Tot_), tidal volume, and instantaneous inspiratory and expiratory airflow throughout the breath cycle. To assess the level of agreement between the XV- and pitot tube-derived measures, which were considered as the reference, bivariate scatter plots, Pearson’s correlation coefficients, and Bland-Altman analyses were used. Mixed effects models were used to characterize the associations between XV- and pitot tube-measurements while accounting for repeated measures resulting from respiratory phase (i.e., inspiratory and expiratory) and mode of ventilation (spontaneous and bi-level) with the study participant as the random effect. Post-hoc estimates of the marginal model adjusted means and effects were used for statistical inference. Furthermore, to account for multiple comparisons, Fischer’s protected least significant different method was used. Finally, the derived mixed models were used to adjust XV-values and predict pitot tube derived values along with the respective 95% limits of agreement. Given that a validation dataset was not available, the bootstrap approach with 1000 iterations was used to assess the robustness of the mixed-effects regression models. All statistical analyses were conducted in Stata 17.0 (StataCorp., College Station, TX) and R version 4.1.2 or above (R Foundation for Statistical Computing), and a p-value of less than 0.05 was used as a threshold for statistical significance [11].

## Results

Twenty-four healthy adults with normal pulmonary function tests, all of whom were lifelong nonsmokers, were assessed with concurrent measurements using the pitot-tube and XV during tidal breathing in the supine position. A subset of 11 healthy adults were reassessed with bilevel positive airway pressure assisted breathing. The mean age and BMI of the sample was 42.8 years (SD: 11) and 24.9 kg/m^2^ (SD: 5.4), respectively and 41% (N = 10) were women (Table 1). The mean FEV_1_ and FVC percent predicted were 96.1% (SD: 14.3) and 97.8% (SD: 13.0), respectively, with no evidence of an obstructive or restrictive ventilatory defect in any of the study participants. Among the participants, the lowest and the highest median Effective Dose (ED) were 0.41 mSv and 0.84 mSv, respectively.

**Table 1:** Sample characteristics

Variable	Mean (SD)		Range
Age, years	42.8	(11.0)	[26.0–63.0]
BMI, kg/m^2^	24.9	(5.4)	[17.9–44.8]
FEV_1_, %	96.1	(14.3)	[74.2–121.0]
FVC, %	97.8	(13.0)	[71.2–124.0]
FEV_1_/FVC, %	97.8	(7.3)	[85.5–111.0]

FEV_1_: Forced expiratory volume in one second percent predicted

FVC: Forced vital capacity percent predicted

**Fig. 2:**
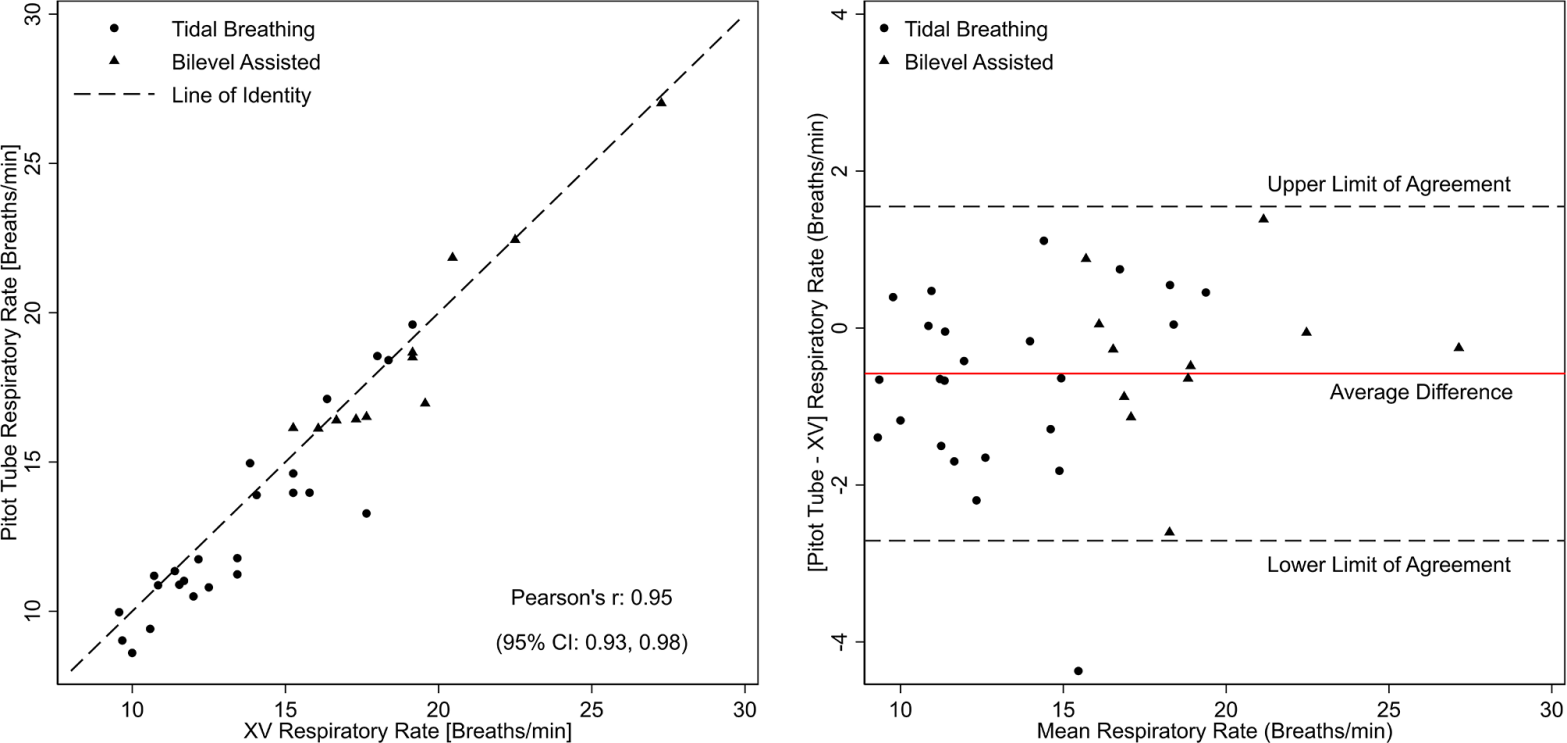
Scatterplot of XV- and pitot tube-derived respiratory rate (left panel) and a Bland-Altman plot with average difference and the corresponding 95% limits of agreement (right panel). Circles and triangles represent values derived from spontaneous (tidal) and bilevel assisted breathing, respectively

**Fig. 3:**
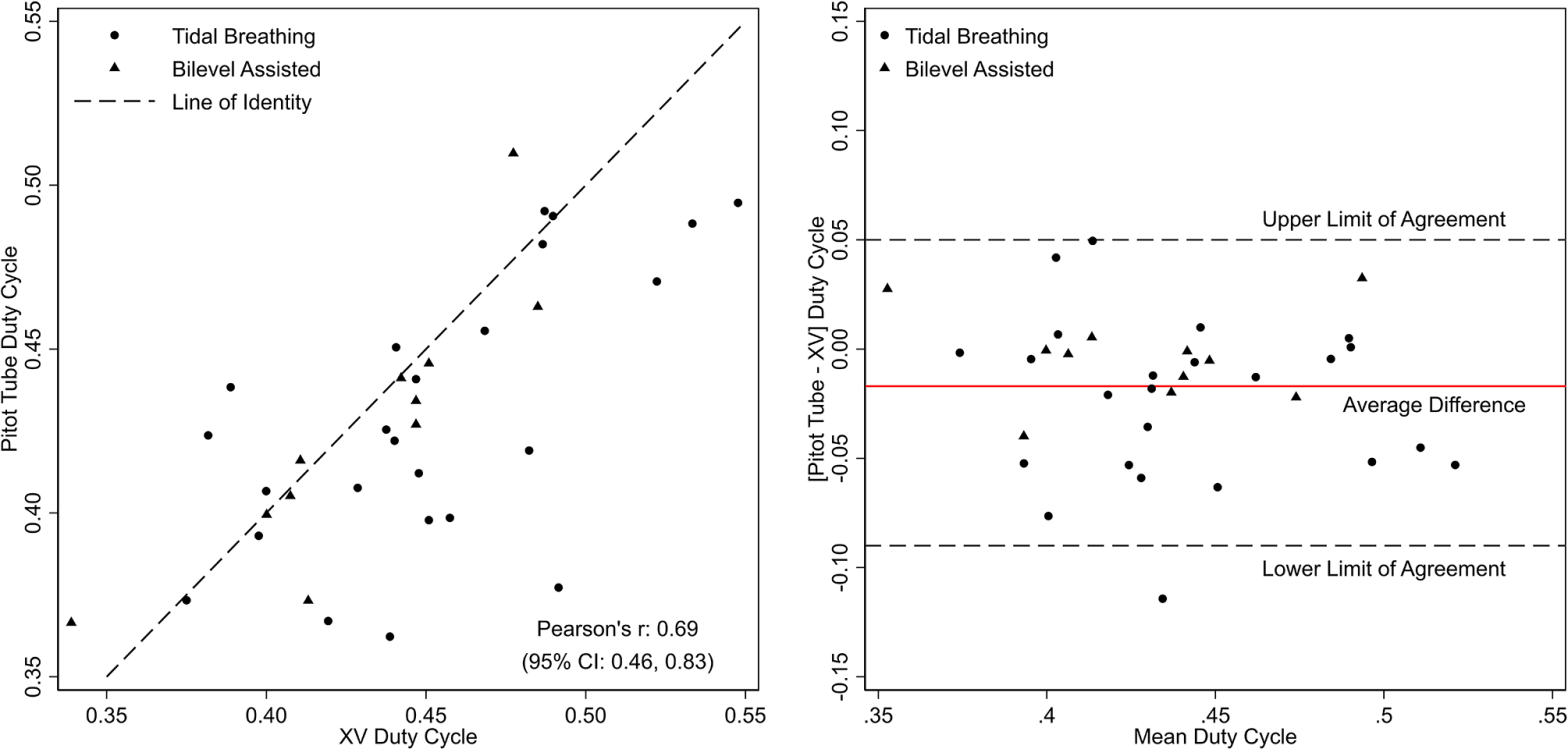
Scatterplot of XV- and pitot tube-derived duty cycle (left panel) and a Bland-Altman plot with average difference and the corresponding 95% limits of agreement (right panel). Circles and triangles represent values derived from spontaneous (tidal) and bilevel assisted breathing, respectively

Table 2 lists the parameters of ventilation derived from XV and pitot-tube, stratified by spontaneous (tidal) and bilevel-assisted ventilation. Figure 2 (left panel) is the scatterplot comparing the respiratory rates between the two methods. While statistically significant differences were noted in respiratory rate, the overall difference was small and clinically insignificant (~ 0.6 breaths/minute). Bland-Altman analysis (Fig. 2, right panel) revealed an average difference of 0.59 (95% CI: -1.67, 2.84) between the two methods. Analyses of duty cycle (T_insp_/T_Tot_) were also conducted to compare measurements from XV and the pitot tube (Fig. 3). The average difference for duty cycle between the two methods was 0.02 (95% limits of agreement: 0.01, 0.03) indicating a high level of agreement. Mixed model analysis of respiratory rate and duty cycle, which accounted for respiratory phase and mode of breathing, showed no statistically significant differences between XV- and the pitot tube-derived measurements across spontaneous and bilevel-assisted ventilation.

**Table 2:** Parameter of ventilation comparing pitot-tube flowmeter to XV

Parameter	Pitot Tube			XV			p-value
	Mean	(95% CI)		Mean	(95% CI)		
Respiratory rate, breaths/min	14.7	(13.6–15.9)		15.3	(14.2–16.5)		0.003
Spontaneous	12.8	(11.5–14.0)		13.5	(12.3–14.6)		0.005
Bilevel	19.0	(16.9–21.1)		19.4	(17.3–21.4)		0.23
Tidal Volume, mL	1030.5	(878.5-1182.5)		574.6	(520.2-629.1)		< 0.001
Spontaneous,	859.3	(707.3-1011.3)		542.2	(495.7-588.7)		< 0.001
Bilevel.	1403.9	(1193.0-1614.8)		645.4	(554.0-736.7)		< 0.001
Average Flow Rate, L/min	26.6	(22.9–30.3)		14.2	(12.9–15.5)		< 0.001
Spontaneous	18.5	(15.1–21.9)		11.5	(10.4–12.6)		< 0.001
Bilevel	44.2	(38.2–50.2)		20.0	(17.3–22.7)		< 0.001
Maximum Flow Rate, L/min	46.6	(40.8–52.4)		26.3	(23.8–28.9)		< 0.001
Spontaneous	31.5	(26.1–36.9)		20.9	(18.6–23.3)		< 0.001
Bi-Level	79.6	(68.5–90.7)		38.2	(32.5–43.8)		< 0.001
Duty cycle (T_insp_/T_Tot_)	0.43	(0.41–0.44)		0.44	(0.43–0.46)		0.002
Spontaneous	0.43	(0.41–0.45)		0.45	(0.43–0.47)		0.002
Bilevel	0.42	(0.39–0.44)		0.42	(0.40–0.44)		0.57

T_insp_ = Inspiratory time; T_Tot_: Total time of respiratory cycle; p- comparing XV to the pitot tube values were derived from mixed model analysis which accounted for the repeated measures over respiratory phase and also mode of ventilation

**Fig. 4:**
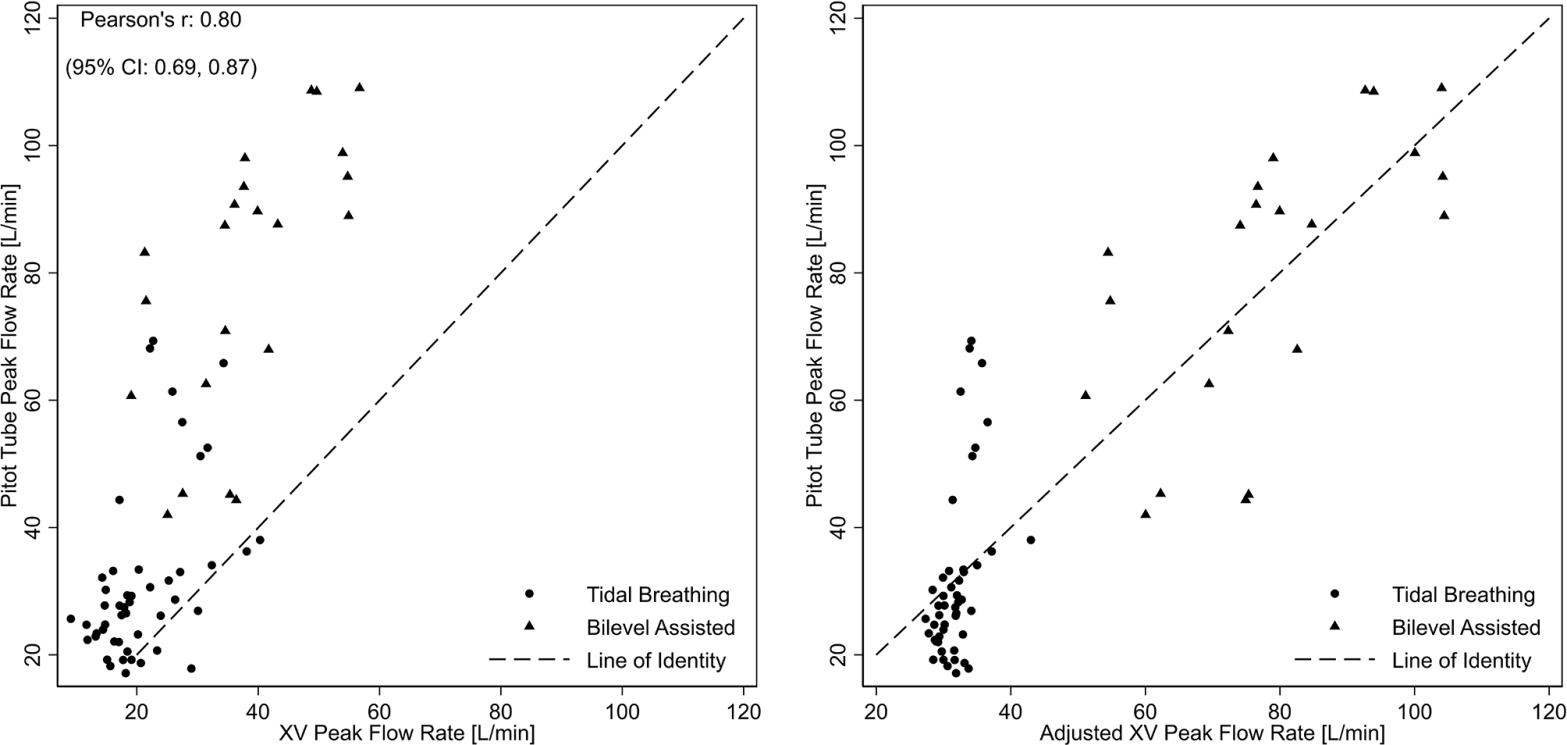
Scatterplot of observed XV- and pitot tube-derived peak flow (left panel) and mixed model adjusted XV-peak flow compared to observed pitot tube-derived peak flow (right panel). Circles and triangles represent values derived from spontaneous (tidal) and bilevel assisted breathing, respectively

**Fig. 5:**
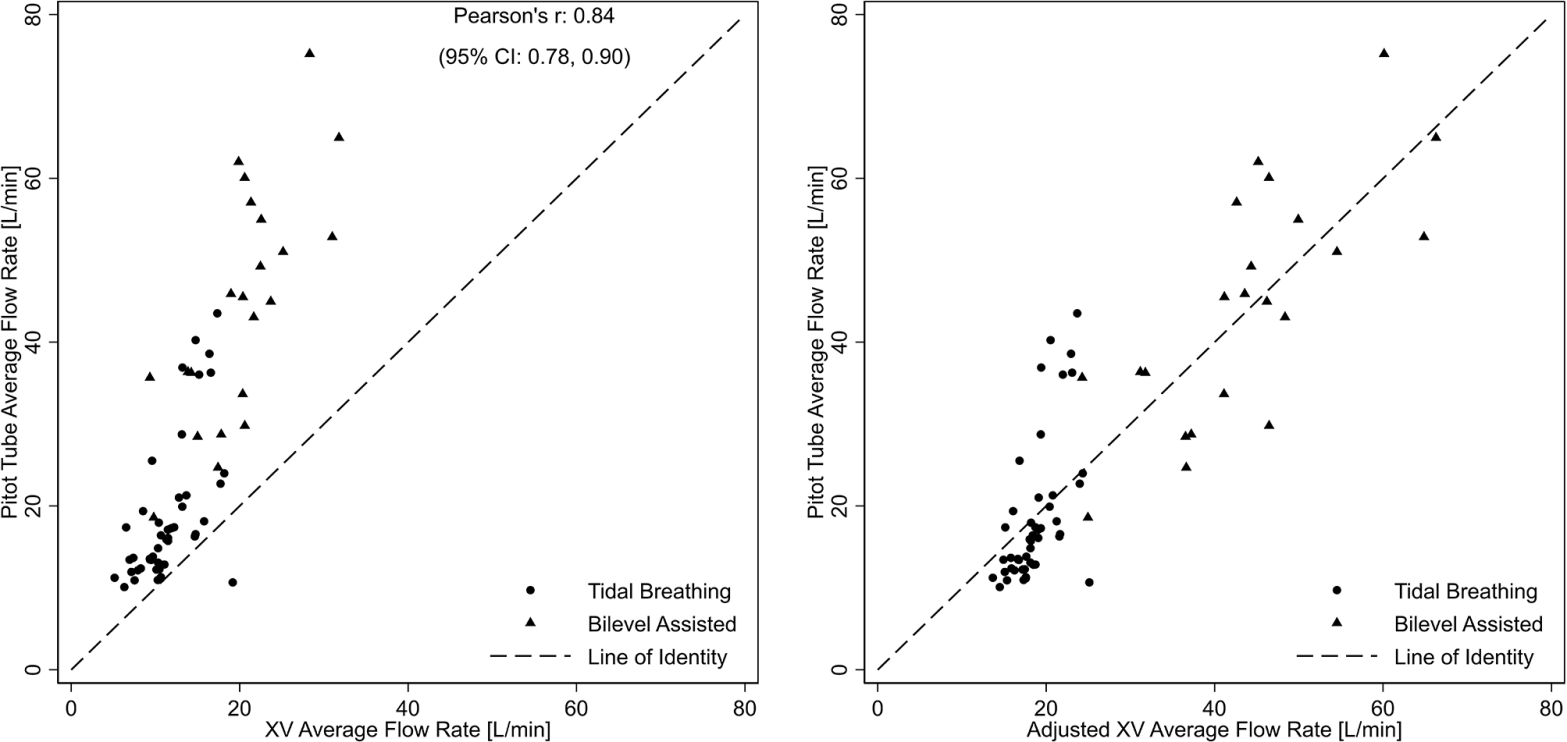
Scatterplot of observed XV- and pitot tube-derived average flow (left panel) and mixed model adjusted XV-average flow compared to observed pitot tube-derived peak flow (right panel). Circles and triangles represent values derived from spontaneous (tidal) and bilevel assisted breathing, respectively

**Fig. 6:**
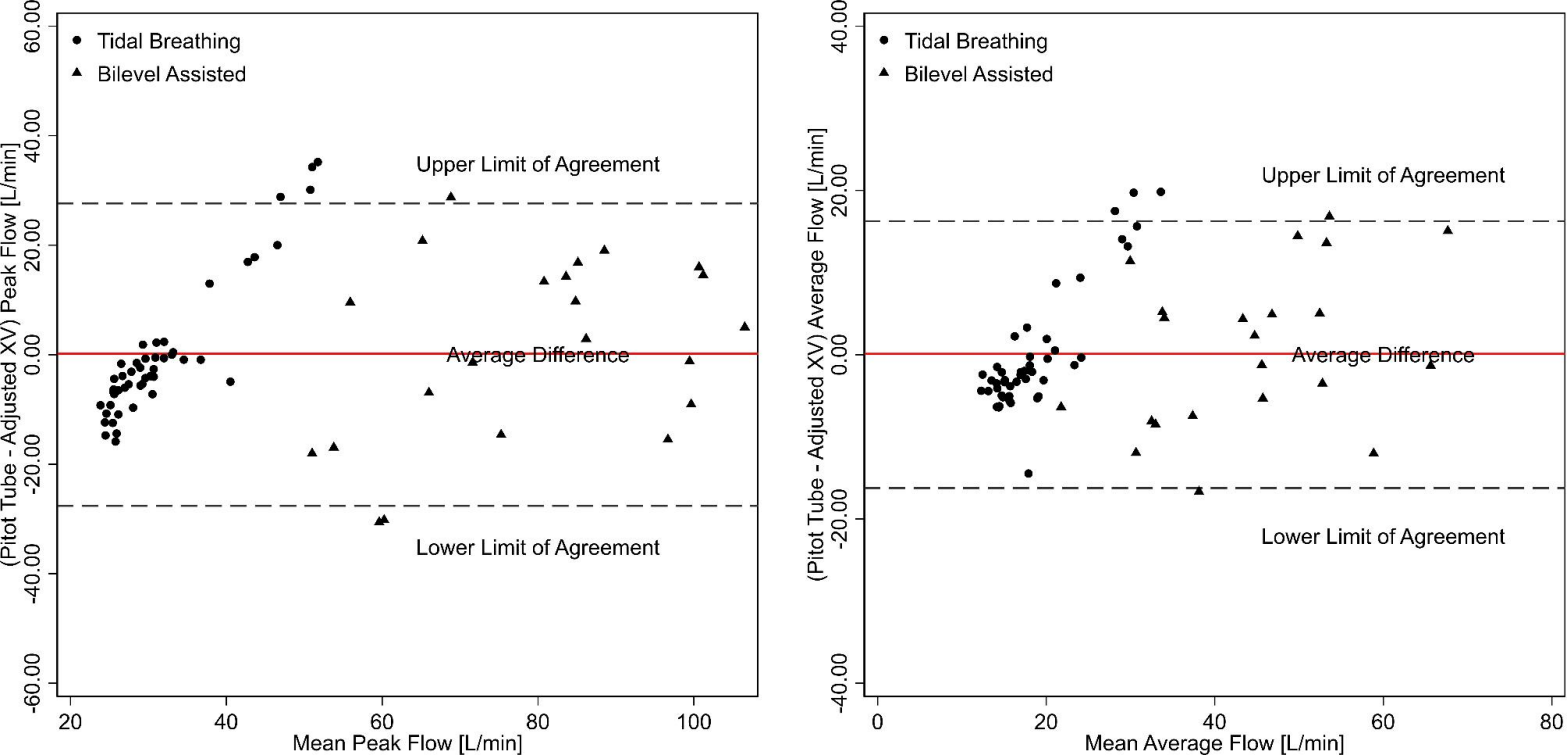
Bland-Altman plot of pitot tube-derived and adjusted XV peak flow (left panel) and average flow (right panel) with mean difference and the corresponding 95% limits of agreement. Circles and triangles represent values derived from spontaneous (tidal) and bilevel assisted breathing, respectively

In contrast to respiratory rate and duty cycle, flow rates (peak and average) were different between the XV- and pitot tube measurements, particularly outside of the normal physiologic flow rates generated during bilevel-assisted breathing (Figs. 4 and 5, left panels). The average difference for peak and average flow rates between XV and pitot tube measures from the Bland-Altman analysis was − 20.3 L/m (95% limits of agreement: -25.3, -15.3) and − 12.4 L/min (95% limits of agreement − 15.7, -9.2), respectively (Fig. 6, left panel). As before, mixed models were used to characterize the association between the pitot tube and the XV-derived flowrates. Using these models, pitot tube flow rates were estimated using the XV data and compared to the observed pitot tube flow rates. As shown in Figs. 4, 5 and 6 (right panels), the mixed model-based adjustment of XV-derived values provide for a higher degree of agreement with pitot-tube derived values, particularly for bilevel-assisted ventilation measurements, which are outside of XV’s typical operating range of tidal ventilation. The bootstrap approach to assessing model fit for peak and average flow showed that there were no failures and the models were convergent indicating model robustness (see supplement for parameters estimates and bootstrap analysis).

**Fig. 7:**
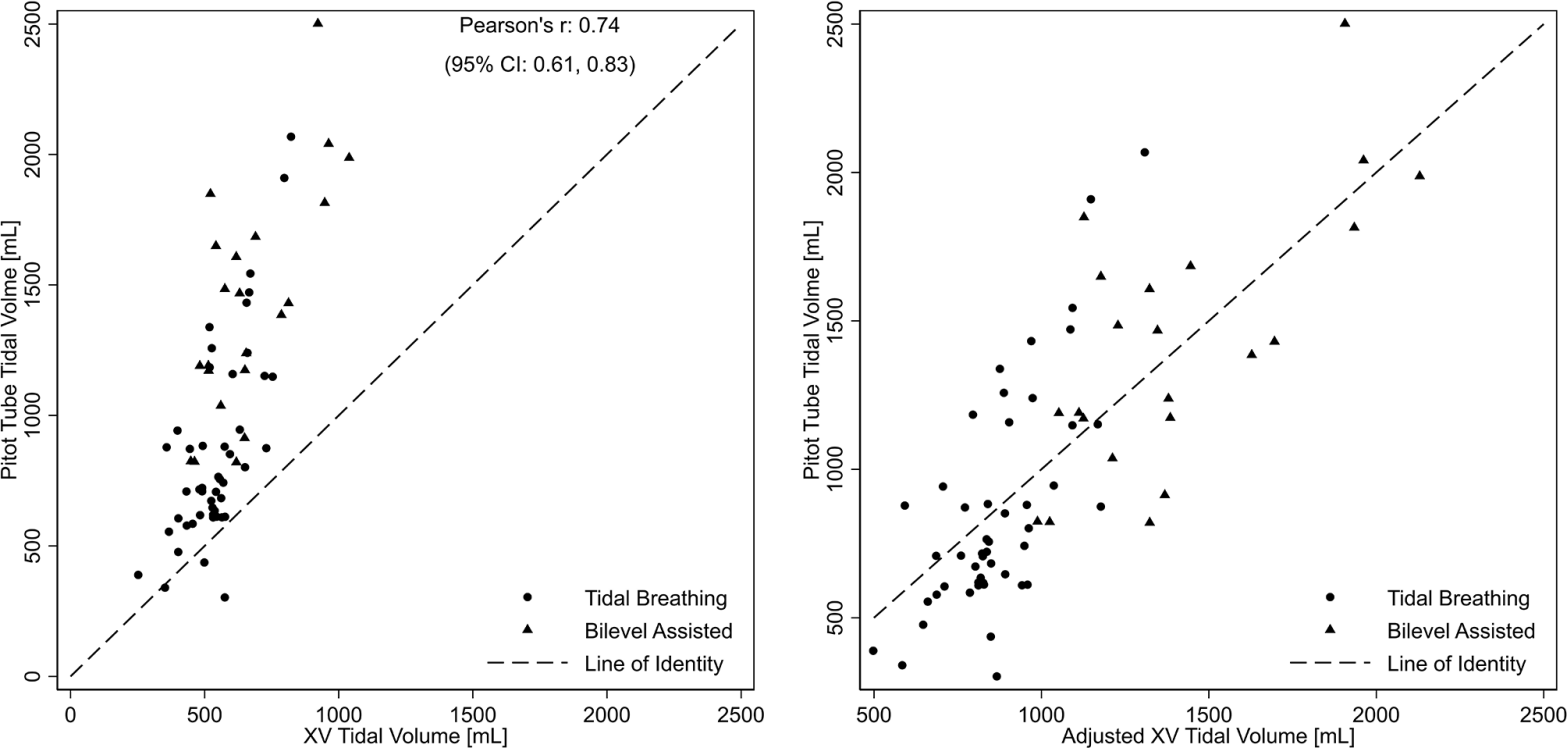
Scatterplot of observed XV- and pitot tube-derived tidal volumes (left panel) along with scatterplot of mixed model adjusted XV-tidal volumes compared to observed pitot tube-derived tidal volumes (right panel). Circles and triangles represent values derived from spontaneous (tidal) and bilevel assisted breathing, respectively

As with airflow measurements, systematic differences were observed between XV- and pitot tube-derived values, particularly for measurements outside the normal physiologic tidal ranges during bilevel-assisted breathing. The average difference for tidal volume from the Bland-Altman analysis was − 455.8 mL (95% limits of agreement: -580.3, -331.4) with XV measurements underestimating the pitot-tube derived values. Because of this systematic difference, a mixed linear model was used to adjust the observed XV tidal volumes. The resulting regression model was used to predict a pitot tube tidal volume using the XV data and compared to the observed pitot tube derived tidal volumes. As shown in Fig. 7 (right panel), the predicted pitot tube tidal volumes from the XV data show a higher degree of agreement than that of the derived from the pitot-tube for all tidal volumes (left panel).

## Discussion

The results of this study demonstrate that parameters of ventilation including respiratory rate, duty cycle, tidal volume, and flow rate assessed with XV show a high degree of correlation with those derived from a pitot tube. Analysis of respiratory rate showed that the difference between the two methods, while statistically different, was not of any clinical significance. For tidal volume and flow rates (inspiratory and expiratory), while there was a high degree of correlation between the two approaches, there was a systematic bias, particularly at higher volumes and flow rates during bilevel-assisted breathing, which are outside of XV’s typical tidal breathing operating range. This bias was adjusted using a model to then predict volumes and flow rates, which were highly correlated with values observed with the pitot tube. The use of such models allows a better optimization of XV-derived tidal volumes and flow rates when XV is utilized outside its typical tidal operating range.

To our knowledge, this is the first study examining the utility of XV in humans to assess dynamic parameters of ventilation. Previously, use of X-ray velocimetry for assessment of lung function has been predominantly limited to preclinical models. 12 In one of its earliest uses, XV was able to identify regional alterations of lung motion in a murine model of bleomycin toxicity corresponding to areas with the most pathological changes induced by bleomycin [6]. Despite the significant heterogeneity of lung injury, XV showed significant differences in regional lung motion well before impairments in global lung compliance. Thus, in that model, XV was able to detect abnormalities that are not evident in global measures of lung function. In addition to detecting regional motion abnormalities associated with alveolar-interstitial injury, XV has also been used to characterize changes in regional changes in airflow. Using βENaC mice, a murine model of cystic fibrosis like lung disease, XV was able to identify regional deficits in airflow resulting from mucus obstruction [9]. As with bleomycin-related changes, areas of decreased ventilation in the βENaC model had corresponding histological areas of mucus plugging in the bronchial tree [9]. Taken together, the preclinical data on XV show that it can be used to detect abnormalities in lung motion caused by pathology of the airways or the alveolar space. The current study adds to the body of evidence on XV by demonstrating its capacity to quantify parameters of ventilation in healthy volunteers.

A key observation in this study was the systematic differences in measurements of tidal airflow and volume between XV and the pitot tube, particularly outside the tidal physiological range. The observed difference between the two is to be expected. Instantaneous airflow through the upper airway, as assessed by the pitot tube, is determined by the intrinsic resistance of the tracheobronchial tree, the elastic load of the lung and the chest wall, and the neural drive to respiratory muscles. In contrast, XV is based on quantifying voxel kinematics that derive ventilation parameters through measuring displacement of lung parenchyma. While XV-derived airflow and volumes were lower with higher tidal volumes, the resulting values were nonetheless highly correlated with the pitot tube with the correlation being greater during spontaneous than with bi-level-assisted breathing. The underlying cause of the larger difference at higher airflows and volumes is likely a result of two factors. First higher flow rates in the pitot-tube may produce a mix of laminar and turbulent flow thereby potentially changing midstream airflow-pressure relationship. Secondly, XV measures tissue motion within a certain volumetric window surrounding the corresponding voxel, and therefore, voxels that move beyond the specified range may be limited to a maximum value of displacement, thereby resulting in an underestimation of actual parenchyma displacement. Additionally, displacement of the lung independent of increased flow at the airway *(e.g.* lung distortion, pendelluft and collateral ventilation*)* can result in overestimation of ventilation. XV is configured for optimal operation during tidal breathing, where occurrence of such measurement errors is minimized. Notwithstanding these issues, a strong correlation between adjusted XV- and pitot-tube derived values is suggestive that XV has value in estimating parameters of ventilation, even outside its optimal operating range with adjustments.

XV has the potential to characterize how tidal volume and flow rate vary across different lobes and segments of the right and left lung with further validation of regional assessments of ventilation heterogenity [13, 14]. Regional partitioning of tidal volume and flow rates has clinical relevance. For example, the adaptive functional alterations of the tracheobronchial tree that occur after segmentectomy or lobectomy [15] cannot be assessed aside from anatomic assessment using computed tomography [16]. Similarly, either lung volume reduction surgery or placement of endobronchial valves for chronic obstructive lung disease can reduce regional air trapping and hyperinflation [17–20]. However, at the present, techniques on evaluating the physiological effects of these procedures are limited to conventional pulmonary function testing [21] or radiographic imaging [22] which do not offer insight on the functional effects (e.g. assessments of flow and volume) at the site of the procedure or in other regions. Furthermore, segmentation of volume and flow has physiological and clinical relevance for examining the effects of positional or progressive conditions such as prone position on lung recruitment and ventilation distribution in spontaneously breathing patients with mild acute respiratory distress syndrome [23]. Other areas of application include characterization of ventilatory abnormalities associated with radiation injury which can extend well beyond the irradiated field [24] Most importantly, given the relative ease of XV measurement, is its application to the understanding of the natural history of lung disease regarding early functional changes, prior to detection on traditional pulmonary function testing, [25] as well as their response to therapy [4].

There are several strengths in the current study. First, the study sample of healthy volunteers included assessment across a wide range of tidal volumes and flow rates using both spontaneous and assisted ventilation. Second, the temporal synchronization of XV data with that obtained from the pitot-tube allowed for a rigorous breath-by-breath analysis of respiratory rate, tidal volume, inspiratory and expiratory flow rates, and duty cycle. Third, although there was a systematic bias at high tidal volumes and flow rates, a model-based adjustment of the XV data provides robust approximations to that derived from the pitot-tube for assisted breathing. Limitations of this study include the possible high errors during bilevel breathing, the fact that matching global parameters does not guarantee the ability to quantify local differences, and remaining questions of how to apply the global adjustment in flow regionally. Nonetheless, the analysis of the fluoroscopic images may be additionally used to provide further lung physiologic properties not currently described such as the expiratory time constant or lung elastic recoil. While the use of a radiographic procedure to portray regional differences in ventilatory function requires streamlining workflows for widespread adoption, the amount of radiation is markedly less than a CT scan which are commonly performed and repeated in patients with acute and particularly chronic lung disease, and which do not provide functional information. Notwithstanding these considerations, it is necessary to build on this study to include patients with a wide array of lung pathologies that encompass obstructive and restrictive ventilatory impairments to help define functional lung imaging’s utility in understanding not just the anatomical, but also the functional phenotype, particularly over time.

### Electronic supplementary material

Below is the link to the electronic supplementary material.

Supplementary Material 1

## Data Availability

Data and materials will be provided upon request.

## References

[1] Fouras A, Dusting J, Lewis R, Hourigan K (2007). Three-dimensional synchrotron x-ray particle image velocimetry. J Appl Phys.

[2] Fouras A, Dusting J, Sheridan J, Kawahashi M, Hirahara H, Hourigan K (2009). Engineering imaging: using particle image velocimetry to see physiology in a new light. Clin Exp Pharmacol Physiol.

[3] Goonan G, Fouras A, Dubsky S (2018). Array-source X-ray velocimetry. Opt Express.

[4] Vliegenthart R, Fouras A, Jacobs C, Papanikolaou N (2022). Innovations in thoracic imaging: CT, radiomics, AI and x-ray velocimetry. Respirology.

[5] Ng I, Paganin D, Fouras A (2012). Optimization of in-line phase contrast particle image velocimetry using a laboratory x-ray source. J Appl Phys.

[6] Fouras A, Allison BJ, Kitchen MJ (2012). Altered lung motion is a sensitive indicator of regional lung disease. Ann Biomed Eng.

[7] Murrie RP, Werdiger F, Donnelley M (2020). Real-time in vivo imaging of regional lung function in a mouse model of cystic fibrosis on a laboratory X-ray source. Sci Rep.

[8] Berg EJ, Robinson RJ. Stereoscopic particle image velocimetry analysis of healthy and emphysemic alveolar sac models. J Biomech Eng 2011;133(6). 10.1115/1.4004251 21744924

[9] Werdiger F, Donnelley M, Dubsky S (2020). Quantification of muco-obstructive lung disease variability in mice via laboratory X-ray velocimetry. Sci Rep.

[10] Kirkness J, Verma M, McGinley B (2010). Pitot-tube flowmeter for quantification of airflow during sleep. Physiol Meas.

[11] Team RC. R: A language and environment for statistical computing. 2013.

[12] Asosingh K, Frimel M, Zlojutro V et al. Preclinical 4-Dimensional Functional Lung Imaging and Quantification of Regional Airflow: A New Standard in Lung Function Evaluation in Murine Models. *American Journal of Respiratory Cell and Molecular Biology* 2022(ja). 10.1165/rcmb.2022-0055MA PMC9564925 35687482

[13] Georgopoulos D, Gomez A, Mink S (1994). Factors determining lobar emptying during maximal and partial forced deflations in nonhomogeneous airway obstruction in dogs. Am J Respir Crit Care Med.

[14] Aliboni L, Tullio M, Pennati F (2022). Functional analysis of the airways after pulmonary lobectomy through computational fluid dynamics. Sci Rep.

[15] Kim SJ, Lee YJ, Park JS (2015). Changes in pulmonary function in lung cancer patients after video-assisted thoracic surgery. Ann Thorac Surg.

[16] Criner GJ, Agusti A, Borghaei H (2022). Chronic obstructive Pulmonary Disease and Lung Cancer: a review for clinicians. Chronic Obstr Pulm Dis.

[17] Brown MS, Kim HJ, Abtin FG (2012). Emphysema lung lobe volume reduction: effects on the ipsilateral and contralateral lobes. Eur Radiol.

[18] Valipour A, Slebos DJ, Herth F (2016). Endobronchial valve therapy in patients with homogeneous Emphysema. Results from the IMPACT Study. Am J Respir Crit Care Med.

[19] Klooster K, Hartman JE, Ten Hacken NHT, Slebos DJ (2017). Improved Predictors of Survival after Endobronchial Valve treatment in patients with severe Emphysema. Am J Respir Crit Care Med.

[20] Criner GJ, Sue R, Wright S (2018). A Multicenter Randomized Controlled Trial of Zephyr Endobronchial Valve Treatment in Heterogeneous Emphysema (LIBERATE). Am J Respir Crit Care Med.

[21] Wanger J, Clausen JL, Coates A (2005). Standardisation of the measurement of lung volumes. Eur Respir J.

[22] Schuhmann M, Raffy P, Yin Y (2015). Computed tomography predictors of response to endobronchial valve lung reduction treatment. Comparison with Chartis. Am J Respir Crit Care Med.

[23] Cornejo RA, Diaz JC, Tobar EA (2013). Effects of prone positioning on lung protection in patients with acute respiratory distress syndrome. Am J Respir Crit Care Med.

[24] Hanania AN, Mainwaring W, Ghebre YT, Hanania NA, Ludwig M (2019). Radiation-Induced Lung Injury: Assessment and Management. Chest.

[25] Jung T, Vij N. Early diagnosis and real-time monitoring of Regional Lung function changes to Prevent Chronic Obstructive Pulmonary Disease progression to severe Emphysema. J Clin Med 2021;10(24). 10.3390/jcm10245811 PMC8708661 34945107

