# Supplementary material for: Quantifying ventilation by X-ray velocimetry in healthy adults
Source: Respir Res. 2023 Aug 30;24:215. doi: 10.1186/s12931-023-02517-z (PMC10469820; doi:10.1186/s12931-023-02517-z)
Supplement: Supplementary file 1 — Supplementary Material 1 [file 12931_2023_2517_MOESM1_ESM.docx]

**ONLINE SUPPLEMENT**

**Quantifying Ventilation by X-ray Velocimetry in Healthy Adults**

Trishul Siddharthan^1^, Kyle Grealis^1^, Jason P. Kirkness^2^, Tamás Ötvös^2^, Darko Stefanovski^3^, Alex Tombleson^1^, Molly Dalzell^1^, Ernesto Gonzalez^1^, Kinjal Bhatt Nakrani^1^, David Wenger^2^, Michael G. Lester^4^, Bradley W. Richmond^4,5,6^, Andreas Fouras^2^, and Naresh M. Punjabi^1^

^1^ Division of Pulmonary, Critical Care, and Sleep Medicine University of Miami, Miami FL

^2^ 4DMedical, Melbourne, Australia

^3^ University of Pennsylvania

^4^ Division of Allergy, Pulmonary, and Critical Care Medicine Vanderbilt University School of Medicine, Nashville, TN

^5^ Department of Veterans Affairs Medical Center, Nashville, TN

^6^ Department of Cell and Developmental Biology, Vanderbilt University, Nashville, TN.

**Running Title:** X-ray Velocimetry and Ventilation

**Corresponding Author:**

Trishul Siddharthan, MD

Division of Pulmonary, Critical Care, and Sleep Medicine

University of Miami, Miller School of Medicine

Division of Pulmonary, Critical Care and Sleep Medicine

1951 NW 7^th^ Ave, Suite 2308

Miami, FL 33136

Phone: 305-243-6388

**SUPPLEMENTARY METHODS**

Twenty-four healthy adults were recruited from the general community. Written informed consent was obtained for all participants and the study was approved by University of Miami IRB (IRB 02010770). To exclude prevalent comorbidity, each participant completed a demographics questionnaire and pulmonary function testing including spirometry, body plethysmography, and measurement of diffusing capacity of the lung.

**Pulmonary Function Testing**

Spirometry was conducted in accordance with the technical standards document developed by the international joint task force appointed by the American Thoracic Society (ATS) and the European Respiratory Society (ERS).^1^ Body plethysmography and diffusing capacity of carbon monoxide were carried out as per the **ATS/ERS taskforce reports for standardization of lung volume testing^2^ and diffusing capacity of the lung^3^, respectively.**

**Pitot Tube Airflow Meter**

Measurement of airflow is typically conducted with a pneumotachograph, which uses a pitot tube principle to determine midstream airflow rate flowing through a wide bore tube. Two pressure ports for measurement are located centerline of the flow tube to measure upstream and downstream pressure. Pneumotachographs, however, can impose some resistance to airflow and/or increased dead space. To alleviate these issues, a low-resistance, low dead space pitot tube system was used in the current study to assess airflow and derive other parameters of ventilation including tidal volume, respiratory rate, and duty cycle. The pitot tube airflow meter used was a lightweight (1.5 g) polyethylene, low dead space (∼10 cm^3^) and resistance validated pneumotachograph.^4^ The flow tube was positioned in line with airflow from the oro-nasal mask (ResMed, San Diego, USA) affixed over the subject nose and mouth. To assess that the mask was leak free and to ensure that all of the inspiratory and expiratory airflow was captured via the pitot tube, the flow tube open to atmosphere was briefly occluded while the participant was requested to perform an inspiration and expiration. The pitot tube pressure sensors were attached to a previously calibrated transducer box, which produces an analogue output signal (0-1 volt range = 0-100 L/min). This analogue signal was connected to the auxiliary input of the digital recording system (Embla, Natus, USA) and displayed during data collection on the polygraphy software application (RemLogic 3.4, Natus, USA). Airflow signal was captured at a 50 Hz sampling rate with no filter settings. Calibration of the airflow was confirmed using two methods: (1) utilizing three simulated breaths via a three-liter syringe, and (2) a steady state flow generated via a CPAP system in line with an external digital flow meter (TSI, USA). The calibration signals for each participant’s recordings were collected prior to commencing with the protocol and data acquisition.

**Bilevel-Assisted Ventilation**

To increase the range of airflow and volume measurements during spontaneous breathing, a subset of individuals had their mask and pitot tube connected to a bilevel pressure device (ResMed, San Diego, USA). In brief, a two-meter tube with an expiratory valve was connected to S8 autoset (ResMed, Australia) in spontaneous-time mode with an expiratory positive airway pressure of 5 cm H_2_O, an inspiratory positive airway pressure of 15 cm H_2_O and a backup rate of eight breaths per minute as a means to reproducibly increase minute ventilation for each individual. At the time of adding the bilevel pressure to the participant, a training session was provided along with time to stabilize to the new baseline breathing volume and flow. The seal between the mask and the face was re-examined to ensure that the system remained leak free. In the case that leak was detected, the head-strap securing the mask was adjusted (either loosened or tightened) so that the features of the mask designed to eliminate leaks are fully functional. Because the mask system completely covered the nose and mouth no instruction was given in relation to the route or timing of breathing.

**X-ray Velocimetry**

**Positioning and Isocenter:** Fluoroscopy imaging was performed while participants were comfortably positioned supine with a natural head position and body straight and aligned caudally on X-ray table of a C-arm fluoroscope (Artis Zeego, Siemens, Germany) with arms positioned out of the target field of view by laying them above the head. The C-arm detector was moved anteriorly over the participants’ chest such that the angular position is perpendicular with the table (0 degrees rotation, posterior-anterior view). Initially, the table was positioned caudally (y-axis) to set the entire lung in field of view from the lung apices to the costophrenic arches of the diaphragm. Once the caudal position of the table was determined the table was not repositioned in the y-axis during the protocol. To set the isocenter for imaging, both the centering beam and low dose scout image the lung field of view was centered laterally (x-axis) so that the sternum and the vertebral column were midline in the image. Once the correct lateral table position was set, the table was not moved in the x-axis for the duration of the protocol. The x-ray detector was rotated either ±90 degrees to the lateral position and a scout image acquired to set the table height to set the anterio-posterior position (z-axis) of the lungs to the center of the image. Finally, once the table height was set, and thus the isometric position of the participants lungs was fixed, the table was not moved during the imaging protocol.

**Imaging Protocol:** Utilizing a cine imaging protocol with the following settings: 81 KV, 6.4 ms pulse width at a radiation dose of 0.080 μGy/fr, five views of fluoroscopic images were captured: 0° PA (Posterior-Anterior axis), ±36° from PA, and ±72° from PA. All the fluoroscopy views had the same center of rotation. Participant Scans were acquired at each of the five views for ~8 seconds, which was sufficient time to capture at least one complete, continuous breath (inspiration/expiration). Automatic Exposure Control (AEC) of the detector on the X-ray system was active to ensure the captured images had the highest level of signal-to-noise ratio independent of participant factors such as size and density. The subject remained in the same supine position for each of the five fluoroscopic imaging sequences. A Geiger counter with an analogue output was connected to the auxiliary input of the digital recording system and recorded at 50 Hz so that the imaging data acquisition period could be precisely synchronized with the airflow data acquisition post-hoc.

**XV Analysis**

Lung tissue displacement is measured by applying three-dimensional particle image velocimetry applied to measure local tissue expansion throughout the lungs over the course of a breath.^5-7^ Lung volume expansion is then used to calculate regional ventilation from approximately 10^4^ adjacent locations (nodes) throughout the lung, equally distributed throughout the measurement field. Each individual node has an x, y and z axis aligned with the caudal-cranial, left-right lateral and inferior-superior axis of the radiograph. Individuals with larger or smaller lungs will have more-or-less nodes, respectively. Ventilation is expressed in dimensionless form as specific ventilation, defined as the change in volume of a lung region since the start of inspiration (ΔV­), normalized by the volume of the same region at end-expiration (V_0_). The specific ventilation measurements for each node loci are captured for the entire image field and the sum of the specific ventilation measurements for the entire lung region is determined to be the change in lung volume. Individual specific ventilation measurements are displayed in a distribution histogram to permit quantile analysis, such as the proportion of lung in low ventilation regions (LVR) or high ventilation regions (HVR).^8^

From the fluoroscopic image sequences, the start and the end of inspiration and expiration are identified by the analysis software from the movement of the image voxels. Each inspiration and expiration were segmented into seven equal parts of the breath cycle and one zero point (yielding a total of 15 points for each breath per participant) for the purpose of performing head-to-head comparison with pitot tube measurements. Airflow and tidal volume measurements were assessed continuously with instantaneous measurements captured at points equally distributed throughout the inspiratory and expiratory phases. The respiratory rate and duty cycle were taken as the average for the breath length per participant during steady state, therefore, each participant had a single value for respiratory rate and duty cycle.

**SUPPLEMENTARY RESULTS**

A mixed-effects regression model was used to correct for the differences in the pitot tube and XV-derived measures for peak flow and average flow. The mixed model for peak flow was parameterized as follows: Pitot Derived-**Peak** Flow = β_0_ + β_jk_(XV-**Peak** Flow) + ε_i_, where i represents the individual observation and j and k are binary variables with j=inspiration (j=1) vs. expiration (j=2) and k=tidal (k=1) vs. bilevel breathing (k=2). β_0_  in this equation represents overall average pitot derived peak flow across inspiration, expiration, tidal and bilevel breathing. β_11_ represents the slope relating the XV-derived peak flow during tidal inspiration to the overall average pitot derived peak flow. β_12_ represents the slope relating the XV-derived peak flow during tidal expiration to the overall average pitot derived peak flow. β_21_ represents the slope relating the XV-derived peak flow during bilevel inspiration to the overall average pitot derived peak flow. β_22_ represents the slope relating the XV-derived peak flow during bilevel expiration to the overall average pitot derived peak flow.

| **Parameter** | **Coefficient (95% CI)** | **Standard Error** | **Z-Score** | **P-value** |
| --- | --- | --- | --- | --- |
| β_0_ | 22.75 (13.62 – 31.87) | 4.65 | 4.89 | <0.001 |
| β_11_ | 0.38 (-0.07 – 0.83) | 0.23 | 1.65 | 0.098 |
| β_12_ | 0.50 (-0.09 – 1.09) | 0.30 | 1.66 | 0.096 |
| β_21_ | 1.43 (1.21 – 1.66) | 0.12 | 12.40 | <0.001 |
| β_22_ | 1.49 (1.25 – 1.72) | 0.12 | 12.37 | <0.001 |

In the absence of a distinct sample to the validate the mixed model, predicted values from the model were then compared back to the pitot tube derived peak flow values. Given that a validation dataset was not available, the bootstrap approach with 1000 iterations was used and it showed that the model goodness of fit was not dependent on a given set of observations. Model parameters and associated errors showed no decrease in the strength of association between XV- and pitot tube-derived peak flow. In addition, in all 1000 iterations, there were no failures and the model was convergent. The following is a Bland-Altman plot relating the corrected XV-based pitot tube estimates to the observed pitot tube measurements of peak flow.

A similar approach was used for the average flow comparing pitot tube and XV-derived measures. As before, the mixed model was parameterized as follows: Pitot Derived-**Average** Flow = β_0_ + β_jk_(XV-**Average**) + ε_i_. The model coefficients for the average flow are as follows.

| **Parameter** | **Coefficient (95% CI)** | **Standard Error** | **Z-Score** | **P-value** |
| --- | --- | --- | --- | --- |
| β_0_ | 9.99 (3.74 – 16.24) | 3.19 | 3.13 | 0.002 |
| β_11_ | 0.79 (0.16 – 1.42) | 0.32 | 2.47 | 0.013 |
| β_12_ | 0.71 (-0.18 – 1.44) | 0.37 | 1.91 | 0.056 |
| β_21_ | 1.77 (1.44 – 2.10) | 0.17 | 10.52 | <0.001 |
| β_22_ | 1.53 (1.06 – 1.99) | 0.24 | 6.43 | <0.001 |

With the bootstrap approach, model parameters and associated errors for average flow showed no decrease in the strength of association between XV- and pitot tube-derived measures. Similarly, in all 1000 iterations, there were no failures and the model was convergent. The following is a Bland-Altman plot relating the corrected XV-based pitot tube estimates to the observed pitot tube measurements of average flow.

**Analyses Stratified by Mode of Ventilation**

The mixed-effects regression model for peak flow and average flow was estimated for peak and average flow separately. The model was parameterized as follows: Pitot Derived-Peak Flow = β_0_ + β_j_(XV-Peak Flow) + ε_i_, where i represents the individual observation and j is a binary variable with j=inspiration (j=1) vs. expiration (j=2). β_0_  in this equation represents overall average pitot derived peak flow across inspiration and expiration. β_1_ represents the slope relating the XV-derived peak flow during tidal inspiration to the overall average pitot derived peak flow. Β_2_ represents the slope relating the XV-derived peak flow during tidal expiration to the overall average pitot derived peak flow.

| Parameter | Coefficient (95% CI) | Standard Error | Z-Score | P-value |
| --- | --- | --- | --- | --- |
| β_0_ | 24.82 (18.6 – 31.1) | 3.19 | 7.77 | < 0.001 |
| β_1_ | 0.28 (0.09 – 0.48) | 0.10 | 2.85 | 0.004 |
| Β_2_ | 0.37 (0.11 – 0.63) | 0.13 | 2.78 | 0.005 |

For bilevel breathing, the mixed-effects regression parameters were as follows:

| Parameter | Coefficient (95% CI) | Standard Error | Z-Score | P-value |
| --- | --- | --- | --- | --- |
| β_0_ | 39.4 (16.8 – 62.0) | 11.5 | 3.42 | < 0.001 |
| β_1_ | 1.06 (0.51 – 1.59) | 0.25 | 4.16 | < 0.001 |
| Β_2_ | 1.05 (0.56 – 1.56) | 0.28 | 3.78 | < 0.001 |

A similar approach was used for the average flow comparing pitot tube and XV-derived measures. As before, the mixed model was parameterized as follows: Pitot Derived-Average Flow = β_0_ + β_j_(XV-Average) + ε_i_. The model coefficients for the average flow during tidal breathing are as follows.

| **Parameter** | **Coefficient (95% CI)** | **Standard Error** | **Z-Score** | **P-value** |
| --- | --- | --- | --- | --- |
| β_0_ | 19.0 (12.6 – 25.2) | 3.21 | 5.88 | < 0.001 |
| β_1_ | 0.98 (0.28 – 1.67) | 0.35 | 2.76 | 0.006 |
| Β_2_ | 1.25 (0.29 – 2.20) | 0.49 | 2.55 | 0.011 |

For bilevel breathing, the mixed-effects regression parameters for average flow were as follows:

| **Parameter** | **Coefficient (95% CI)** | **Standard Error** | **Z-Score** | **P-value** |
| --- | --- | --- | --- | --- |
| β_0_ | 24.5 (2.8 – 46.3) | 11.1 | 2.21 | < 0.001 |
| β_1_ | 1.06 (1.8 – 3.4) | 0.41 | 6.26 | < 0.001 |
| Β_2_ | 1.05 (1.9 – 4.0) | 0.52 | 5.62 | 0.027 |

**Radiation Dose Measurement**

The effective dose (ED) associated with the XV lung imaging protocol was determined. To calculate ED, Dose Area Product (DAP) data was obtained from the metadata taglines of the fluoroscopic DICOM images. From the DAP, an ED in mSv was estimated using the Dose Conversion Coefficient from the National Council on Radiation Protection and measurements.^9^ A reasonable coefficient was taken to be 0.12 on the low end and 0.26 on the high end. The final ED estimates for the 8 sec imaging protocol for each participant was derived. Among the 24 participants, the lowest and the highest median Effective Dose (ED) were 0.41 mSv and 0.84 mSv, respectively. These estimates equate to approximately four to nine chest X-rays, respectively. The ED is directly related to participant size, habitus and relative composition, such that a smaller total body size and less dense tissue composition result in lower value.

**References**

1. Graham BL, Steenbruggen I, Miller MR, et al. Standardization of spirometry 2019 update. An official American thoracic society and European respiratory society technical statement. *American journal of respiratory and critical care medicine.* 2019;200(8):e70-e88.

2. Wanger J, Clausen J, Coates A, et al. Standardisation of the measurement of lung volumes. *European respiratory journal.* 2005;26(3):511-522.

3. Graham BL, Brusasco V, Burgos F, et al. 2017 ERS/ATS standards for single-breath carbon monoxide uptake in the lung. *European Respiratory Journal.* 2017;49(1).

4. Kirkness J, Verma M, McGinley B, et al. Pitot-tube flowmeter for quantification of airflow during sleep. *Physiological measurement.* 2010;32(2):223.

5. Fouras A, Allison BJ, Kitchen MJ, et al. Altered lung motion is a sensitive indicator of regional lung disease. *Annals of biomedical engineering.* 2012;40(5):1160-1169.

6. Dubsky S, Hooper SB, Siu KK, Fouras A. Synchrotron-based dynamic computed tomography of tissue motion for regional lung function measurement. *Journal of The Royal Society Interface.* 2012;9(74):2213-2224.

7. Stahr CS, Samarage CR, Donnelley M, et al. Quantification of heterogeneity in lung disease with image-based pulmonary function testing. *Scientific reports.* 2016;6(1):1-10.

8. Eddy RL, Svenningsen S, McCormack DG, Parraga G. What is the minimal clinically important difference for helium-3 magnetic resonance imaging ventilation defects? *European Respiratory Journal.* 2018;51(6).

9. National Council on Radiation Protection and Measurements. Ionizing radiation exposure of the population of the United States. 2009.
